# Proteomic Profile Distinguishes New Subpopulations of Breast Cancer Patients with Different Survival Outcomes

**DOI:** 10.3390/cancers15174230

**Published:** 2023-08-24

**Authors:** Joanna Tobiasz, Joanna Polanska

**Affiliations:** 1 Department of Data Science and Engineering, Silesian University of Technology, 44-100 Gliwice, Poland; joanna.tobiasz@polsl.pl; 2 Department of Computer Graphics, Vision and Digital Systems, Silesian University of Technology, 44-100 Gliwice, Poland

**Keywords:** breast cancer, machine learning, survival analysis, subtyping, survival analysis, effect size

## Abstract

**Simple Summary:**

Breast cancer is a heterogeneous disease, and several attempts have been made to subtype it. Based on the abundance of 86 proteins, we identified six subpopulations of breast cancer patients: one basal subtype, one HER2-enriched subtype, and four luminal subtypes. We then evaluated them demographically and clinically, and we found significant differences in survival. The new luminal subpopulations vary in prognoses. One, marked as A2, showed similar or even worse survival than HER2-enriched and basal cases. The observed poorer survival cannot be explained by clinical and demographic factors, as there was no substantial association with race, ethnicity, metastasis, tumor size, cancer stage, and age at diagnosis. It suggests that the molecular profiles underlie the cohort heterogeneity rather than the patient background. The subpopulations identified may potentially complement established breast cancer classifications and, with further molecular investigation, may find application in clinical routine.

**Abstract:**

As a highly heterogeneous disease, breast cancer (BRCA) demonstrates a diverse molecular portrait. The well-established molecular classification (PAM50) relies on gene expression profiling. It insufficiently explains the observed clinical and histopathological diversity of BRCAs. This study aims to demographically and clinically characterize the six BRCA subpopulations (basal, HER2-enriched, and four luminal ones) revealed by their proteomic portraits. GMM-based high variate protein selection combined with PCA/UMAP was used for dimensionality reduction, while the k-means algorithm allowed patient clustering. The statistical analysis (log-rank and Gehan–Wilcoxon tests, hazard ratio HR as the effect size ES) showed significant differences across identified subpopulations in Disease-Specific Survival (*p* = 0.0160) and Progression-Free Interval (*p* = 0.0264). Luminal subpopulations vary in prognosis (Disease-Free Interval, *p* = 0.0277). The A2 subpopulation is of the poorest, comparable to the HER2-enriched subpopulation, prognoses (HR = 1.748, referenced to Luminal B, small ES), while A3 is of the best (HR = 0.250, large ES). Similar to PAM50 subtypes, no substantial dependency on demographic and clinical factors was detected across Luminal subpopulations, as measured by χ^2^ test and Cramér’s V for ES, and ANOVA with appropriate post hocs combined with *η^2^* or Cohen’s d-type ES, respectively. Progesterone receptors can serve as the potential A2 biomarker within Luminal patients. Further investigation of molecular differences is required to examine the potential prognostic or clinical applications.

## 1. Introduction

Breast cancer is a highly heterogeneous disease, demonstrating diverse molecular and histological backgrounds and varying clinical outcomes. The well-recognized clinical classification of breast cancer is based on several marker genes and proteins, mainly estrogen receptors (ER), progesterone receptors (PR), and human epidermal growth factor receptor 2 (HER2). Despite several limitations and imperfect representation of molecular profiles, clinical classification has remained unmodified for years [1,2].

In the early 2000s, gene expression profiling allowed the identification of five intrinsic molecular subtypes, which was partly inconsistent with the clinical classification [3,4,5]. Following those findings, Parker et al. [6] proposed a 50-gene Prediction Analysis of Microarray (PAM50), which aimed to predict the chemotherapy benefit and breast cancer prognosis and allowed the intrinsic subtype diagnosis for five molecular subtypes: basal-like, HER2-enriched, luminal A, luminal B, and normal-like. The last one is currently considered an artifact resulting from the contamination of tumor samples with normal breast tissue [2].

Nonetheless, multiple mechanisms impact the gene expression between transcriptomic and proteomic layers, while the advances in high-throughput technologies currently allow expression investigation beyond the transcriptomic level. Many attempts for breast cancer subtyping are hence proposed to retrieve a more comprehensive insight into disease stratification. Various approaches for hierarchical clustering were used for the subgroup identification [3,5,6,7,8,9,10,11,12]. In some studies, k-means clustering or Gaussian mixture models (GMM) were also applied [12,13]. Moreover, several state-of-the-art approaches for multimodal subtyping have been proposed lately [14,15,16,17]. In most of the studies, the results of subtyping are evaluated with the survival analysis, mainly based on the Kaplan–Meier (KM) graphs, log-rank test, and Cox proportional hazard model [8,9,10,11,12,13,14,15,16,17]. Numerous studies investigated the results with regard to demographic background or clinical metadata, like cancer stage, metastasis, tumor size, or nodes affected [8,10,12,15].

This study aimed to reveal and evaluate breast cancer patient subpopulations identified based on proteomic profiles. Using dedicated statistical analysis appropriate for challenging and imbalanced data sets, we investigated the revealed subpopulations regarding their clinical experience and demographic background. The purpose was to verify if the heterogeneity in the breast cancer cohort can be explained by the patient background diversity or is rather caused by molecular factors undetected at the transcriptomic level. Apart from basal and HER2-enriched subpopulations, we identified four luminal ones demonstrating significant differences in survival. Revealed subpopulations did not show a larger dependency on the demographic or cancer staging factors than the original PAM50 classification. Therefore, we suppose the re-identifying breast cancer subpopulations may additionally represent previously hidden sources of tumor diversity and potentially complement the existing subtyping approaches.

## 2. Materials and Methods

### 2.1. Materials

The data sets used for this study were collected as a part of the TCGA Breast Invasive Carcinoma (TCGA-BRCA) project. The data files were downloaded from the Genomic Data Commons (GDC) Data Portal [18] or Legacy Archive [19], depending on the file type. We only considered the primary tumor samples from female donors.

The protein levels were measured with the Reverse Phase Protein Array (RPPA) platform and acquired from GDC in the preprocessed version following the correction for loadings and median-centering across antibodies [20,21]. We considered only the records for 166 proteins with no missing values within the cohort. Moreover, the subtype labels from the 50-gene PAM50 predictor [6] used in this study were published by The Cancer Genome Atlas Network [7].

The demographic information provided by TCGA Research Network included age at the initial diagnosis, declared race, and ethnicity. The available clinical data contained the vital status, time from the initial diagnosis to the last contact with a patient, and, in the case of a patient’s death, the time survived from the initial diagnosis, and the American Joint Committee on Cancer (AJCC) cancer staging fields of tumor T (describing the size of the tumor and its spread to the neighboring tissues), regional nodes N (defining cancer spread to nearby lymph nodes), metastases M (denoting if cancer passed on to other parts of the body), and stage. Moreover, information on whether a patient received radio-, chemo-, or hormone therapy was provided. For the survival analysis, TCGA Pan-Cancer Clinical Data Resource (TCGA-CDR) dataset was used, which was curated, standardized, and described by Liu et al. [22].

We checked the protein level data set for the batch effect. For the data dimensionality reduction, we applied the Uniform Manifold Approximation and Projection (UMAP) algorithm [23] to the data space reduced with the Principal Components Analysis (PCA) [24], consisting of top PCs explaining 90% of the variance in the data. The Euclidean distance served as the similarity measure. We visually verified the existence of the technical bias by checking if the samples collected at the same medical center or assigned to the same plate would group at the UMAP embedding. We detected a batch effect due to the study design, and we corrected it with ComBat algorithm included in the “sva” R package [25]. Following the batch effect correction, samples with unknown PAM50 labels were excluded from the study. Moreover, the records for the five patients classified by the PAM50 predictor as normal-like subtypes were rejected due to their low representation and recognition of this subtype as an artifact [2]. The final data set included the measurements for 407 patients.

### 2.2. Identification of Patient Subpopulations

The protein levels measurements served for the identification of breast cancer patient subpopulations. Firstly, we performed the feature filtration based on the GMM. The log2-scaled variances of protein levels were GMM decomposed for the number of components from 1 to 5 chosen based on the Bayesian Information Criterion (BIC) [26] as described by Marczyk et al. [27]. The crossing points of the top two components corresponding to the highest variance served as the filtration threshold. We selected the optimal number of clusters with the GAP criterion [28]. A comparison of other combinations of feature engineering and clustering methods, as well as the optimization of their parameters and quality assessment using the proposed metrics, have been described previously [29].

We related the revealed subpopulations to the transcriptomics-based PAM50 labels. Furthermore, we compared the obtained results with the clustering outcomes of a similar procedure performed on the RNA-sequencing (RNA-seq) measurement for part of the TCGA-BRCA cohort [30]. Figure S1 presents the overlapping of patients between the data sets of all available PAM50 labels, RPPA-based protein levels, and RNA-seq measurements used by Tobiasz et al. [30].

### 2.3. Clinical Characteristics of Patient Subpopulations

We evaluated both our proposed subpopulations of breast cancer patients and PAM50 subtypes by investigating individuals’ clinical and demographic profiles. We aimed to verify whether the survival and clinical experiences or the demographic background carry any differentiating significance and support the protein-based detection of subpopulations.

#### 2.3.1. Survival Analysis

We investigated four clinical survival outcome endpoints: Overall Survival (OS), Disease-Specific Survival (DSS), Disease-Free Interval (DFI), and Progression-Free Interval (PFI), as defined by Liu et al. [22]. However, OS and DSS are not recommended for the TCGA-BRCA project due to a short-term follow-up interval, so the results must be considered with caution.

We used the survival function’s KM estimator to plot the survival curves for the identified subpopulations [31]. We assessed the differences in survival between the groups with log-rank and Gehan–Wilcoxon tests. The rationale for using both approaches is that while the log-rank test puts the same importance on differences between the survival functions throughout the whole timespan of the study, the Gehan–Wilcoxon test emphasizes the early changes in survival when the sample size is still relatively large [32,33,34,35,36]. Moreover, we fitted the Cox proportional hazard models to estimate the hazard ratio (HR) corresponding to each subpopulation compared to the one defined as the reference [37]. The thresholds for HR interpretation were adjusted for the imbalance between the sizes of the compared subpopulations, represented by so-called allocation probability [38,39].

#### 2.3.2. Demographic and Clinical Profiles

Multiple AJCC Cancer Staging Manual editions (mainly the 6th and 7th ones released in 2002 and 2010, respectively) served for staging the TCGA-BRCA cohort. Furthermore, for some patients, the edition was unknown and indeterminable based on the records. The Cancer Genome Atlas Network attempted to convert all older versions to the 7th edition of the staging manual and published the results [7]. Hence, in this study, we used the updated stage records when available. For the unconverted cases, we accepted the original cancer stage provided in the TCGA-BRCA.

Due to the insufficient sample size per group, we ignored the highly detailed staging information (like the division into stages IIIA, IIB, and IIIC). Instead, we merged the stages into the following categories: Stage I, Stage II, Stage III, and Stage IV. Moreover, for the same reason, we binarized pathologic fields connected with tumor size (T) and spread to lymph nodes (N), as recommended [7]. The third pathologic field, M, was originally binary, corresponding to metastasis or lack of it. Tumors were denoted as T1 when smaller than 2 cm and “other” otherwise. N was coded as negative if no spread was observed and positive otherwise.

To verify the association between each of the demographic or clinical categorical factors and analyzed subpopulations, we conducted the Pearson χ^2^ test of independence. For the two groups and two categories’ cases (2-by-2 contingency tables), we applied Yates’s correction for continuity [40]. However, as the contingency tables varied in dimensions between testing scenarios due to different numbers of both categories and subpopulations compared, the Pearson χ^2^ test *p*-value failed to provide a good characterization of dependency. Hence, to reliably compare the dependency for proposed subpopulations and PAM50-based subtypes, we calculated Cramér’s V effect size to assess the strength of the association [41]. We adapted the thresholds for Cramér’s V interpretation from Cohen’s *w* cut-off values, depending on the smaller dimension of the contingency table [39].

For the patient’s age at diagnosis, we applied Shapiro–Wilk [42] and Bartlett [43] tests to confirm normality and variance homogeneity, respectively. As both population normality and variance homogeneity assumptions were fulfilled, we compared the age at diagnosis between the subpopulations with a t-test when two groups were considered or a one-way Analysis of Variance (ANOVA) otherwise. Additionally, we calculated the appropriate effect size estimate, depending on the test (Cohen’s d for two samples and $\eta^{2}$ for more than two samples) [39].

### 2.4. Molecular Characteristics of Luminal Patient Subpopulations

The final step was the comparative analysis of protein abundance levels in the identified luminal-type patient subpopulations. We used the Kruskal–Wallis test and the non-parametric modification of the *η*^2^ effect size measure [44], as the normality assumption was not met. We identified the proteins with at least a large effect size (*η*^2^ ≥ 0.14) [39] and investigated the Kyoto Encyclopedia of Genes and Genomes (KEGG) [45] and Reactome [46,47] pathways in which they participate.

## 3. Results

**Figure 1:**
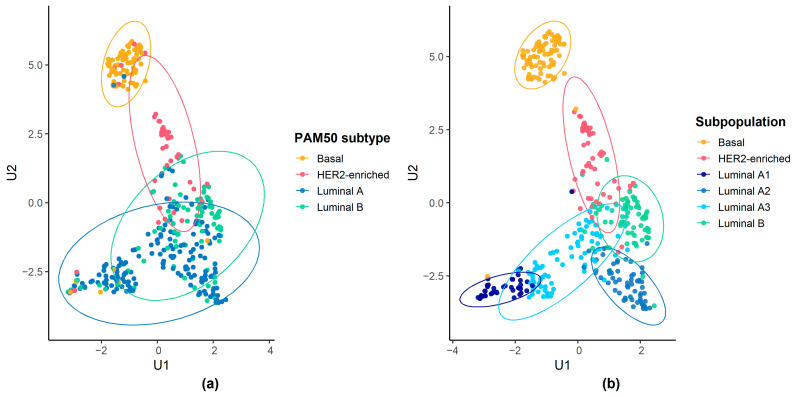
Protein-level-based UMAP visualization with data points colored with regard to breast cancer subtypes. (**a**) Breast cancer subtypes obtained with the PAM50 transcriptomic-based predictor. (**b**) Proposed breast cancer patients’ subpopulations identified with k-means clustering of protein levels preceded by Gaussian-mixture-model-based feature filtration.

GMM-based feature filtration provided the set of 86 proteins used for subpopulation identification. They are listed in Table S1. K-means clustering preceded by GMM-based feature filtration allowed us to distinguish six clusters, which we matched to the available PAM50 etiquettes and named according to the dominant PAM50 subtype. The results of patient subpopulations’ identification are referred to as the PAM50 subtypes in terms of the number and percentages of cases in Table 1. Figure 1 shows UMAP embedding colored with regard to the revealed subpopulations or PAM50 labels. One cluster per basal, HER2-enriched, and luminal B subtypes was identified. Interestingly, three newly discovered clusters corresponded to the luminal A PAM50 subtype (named luminal A1, A2, and A3). This suggests the luminal breast cancers in the TCGA cohort are highly heterogenous and may be more specifically divided into four subpopulations in total instead of two luminal PAM50 subtypes. Hence, we further focused on the investigation of the clinical and molecular differences between those luminal subpopulations as they are the main modification compared to the subtyping by the PAM50 transcriptomic-based classifier.

In Table S2 and Figure S2, the patient subpopulations identified based on the proteomics data are related to the clustering outcomes obtained in a similar study based on RNA-seq results [30]. Table S3 shows RNA-seq-based clustering results compared to the PAM50 subtypes. Furthermore, Figure S3 shows UMAP embedding colored with regard to PAM50 subtypes or the RNA-seq-based clusters.

### 3.1. Clinical Characteristics of Patient Subpopulations

We investigated patients’ clinical and demographic profiles to evaluate the discovered subpopulations, in particular the luminal ones, compared to PAM50 subtypes.

#### 3.1.1. Survival Analysis

**Figure 2:**
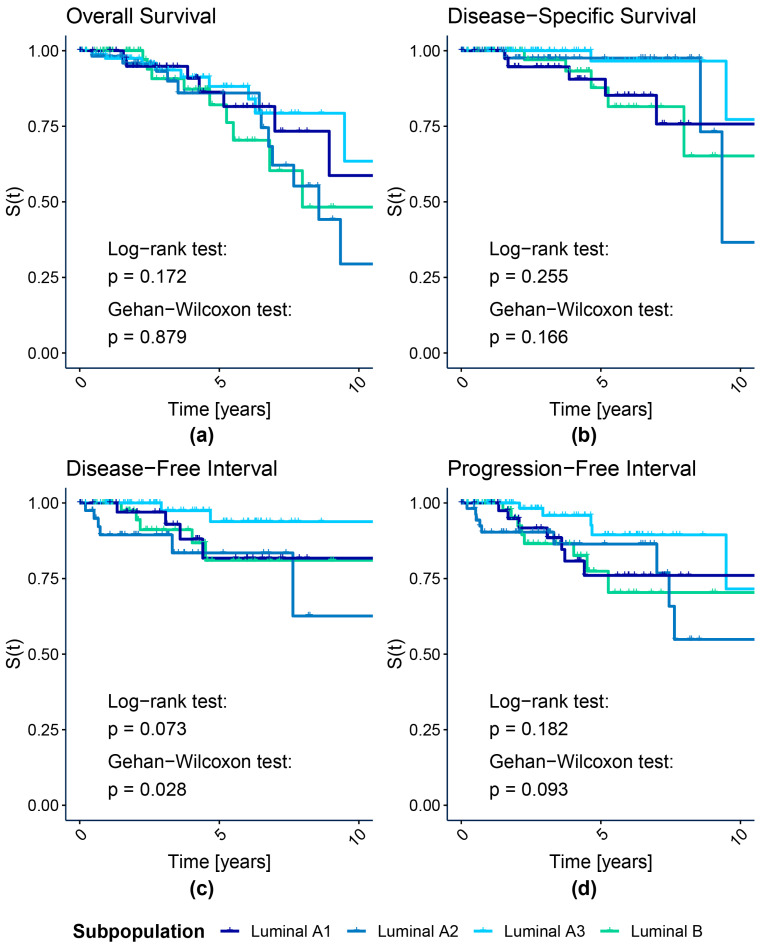
Kaplan–Meier survival curves of luminal subpopulations identified with k-means clustering of protein levels. (**a**) Overall Survival; (**b**) Disease-Specific Survival; (**c**) Disease-Free Interval; (**d**) Progression-Free Interval.

**Figure 3:**
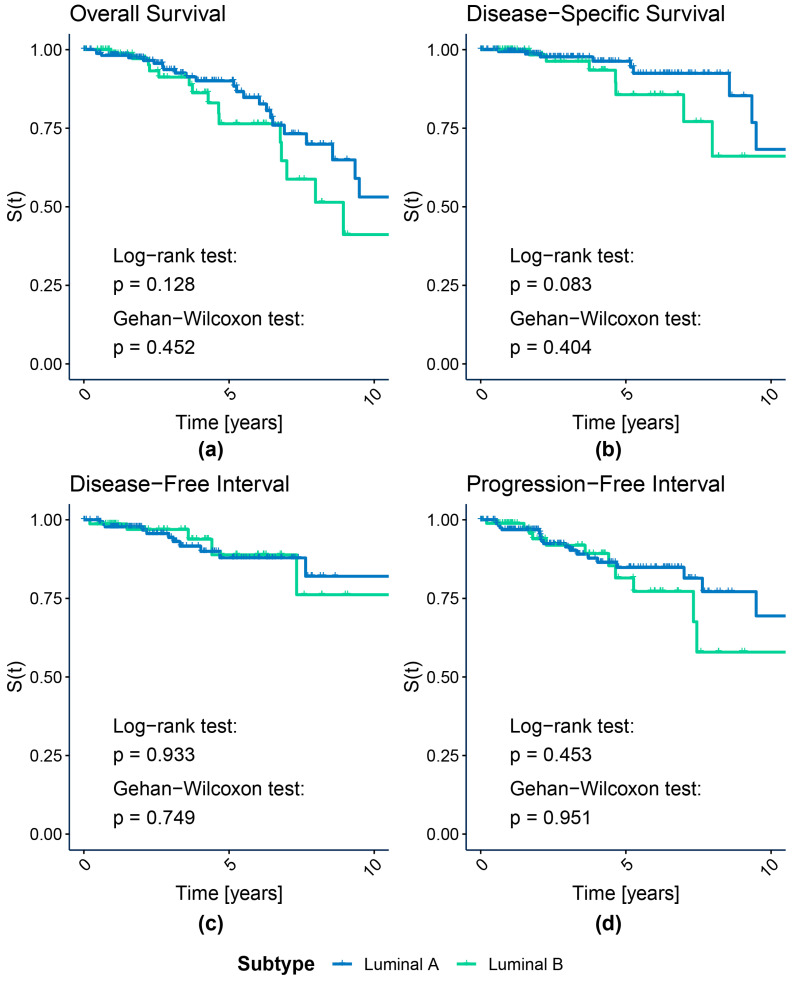
Kaplan–Meier survival curves of luminal PAM50 subtypes. (**a**) Overall Survival; (**b**) Disease-Specific Survival; (**c**) Disease-Free Interval; (**d**) Progression-Free Interval.

Figure 2 shows the KM graphs with log-rank and Gehan–Wilcoxon tests *p*-values for all four luminal subpopulations identified with our k-means procedure. For comparison, we present the analogous graphs for luminal A and B PAM50 subtypes in Figure 3. We highlight here the luminal cases to examine the key modification between our and PAM50-based subtyping approaches, while the remaining HER2-enriched and basal subtypes were highly concordant for both proteomic and transcriptomic subtyping. Nonetheless, the KM graphs for all subpopulations, including HER2-enriched and basal cases, are shown in Figures S4 and S5 for our and PAM50 assignments, respectively. All survival curves were truncated at a 10-year follow-up time for clarity, as suggested by Liu et al. [22], since regardless of the endpoint, few events of interest had been recorded after that time for the TCGA-BRCA cohort. Tables S4 and S5 present the test statistics and *p*-values for k-means- and PAM50-based luminal and all subtypes, respectively.

The luminal subpopulations discovered with k-means differed in DFI, as shown by the Gehan–Wilcoxon test (*p* = 0.0277). No significant survival differences between luminal A and B PAM50 subtypes were discovered, regardless of the test and the endpoint. Interestingly, for the Gehan–Wilcoxon test, which emphasizes the early changes in survival, the *p*-value was lower than for the log-rank test for all endpoints but OS, which is the most biased endpoint among all considered. However, we observed this only for k-means-based luminal subpopulations.

We noticed a distinct drop in DFI and PFI for luminal A2 cases during the first year of follow-up (Figure 2c,d). Two groups similar in DSS are visible in Figure 2b: A2 and A3 luminal subpopulations with a better prognosis and A1 and B luminal subpopulations with worse outcomes. KM graphs indicate that the luminal A3 subpopulation generally shows the best prognosis regarding recurrence among all investigated.

Results of the Cox regression analysis on the detected luminal subpopulations are presented in Table 2. We detected only small or neglectable effects for A1 and A2 subpopulations with reference to luminal B. In concordance with the KM-based observations, the medium and large effect was, however, revealed in favor of luminal A3 tumors, depending on the endpoint. The luminal A2 subpopulation showed a slightly worse survival outcome than luminal B, unlike the other two luminal A subpopulations. Only in terms of DSS was the risk for all luminal A subpopulations lower than for luminal B.

The results of the analogous Cox proportional hazard analysis results performed for PAM50 luminal subtypes are presented in Table 3. The luminal B group served as the reference. In the case of this comparison, we only observed a small effect in terms of OS and PFI and a medium effect for DSS. The hazard was lower for the luminal A group for all endpoints but DFI.

Tables S6 and S7 show analogous results of the Cox proportional hazard analysis presented for all proteomic-based and PAM50 subtypes, respectively, including basal and HER2-enriched. Basal subtypes were used as a baseline.

When we also considered HER2-enriched and basal proteomic-based subpopulations, the *p*-value of the Gehan–Wilcoxon test remained lower than that of the log-rank test for all endpoints but OS (Table S5). Only DSS and PFI differed significantly between the groups. Both basal and HER2-enriched cases showed distinctly poorer OS and DSS than luminals (Figure S4a,b). However, for PHI and DFI recommended for breast cancer, the luminal A2 subpopulation’s outcome was even worse than for HER2-enriched and basal ones, especially shortly after the initial diagnosis (Figure S4c,d).

In Cox regression analysis performed on all revealed subpopulations, we observed small or even neglectable effects for all subpopulations referred to basals, apart from the luminal A3, for which the effect was large per each endpoint but OS, indicating improved prognosis. Regardless of the endpoint type, we noted an increased risk for HER2-enriched subpopulation in reference to basals. For DFI, we also observed a higher risk for the luminal A2 subpopulation. Interestingly, in terms of OS, HR was lower than 1 for the luminal A3 subpopulation only, which seems surprising, as basal tumors are widely considered aggressive and are associated with poor clinical outcomes. This appears to confirm the limited OS reliability.

When we included all PAM50 subtypes in the comparison, Gehan–Wilcoxon *p*-values were lower than their log-rank counterparts for all considered endpoints, indicating the early changes in survival. The differences were statistically significant for OS, DSS, and PFI (Table S5). KM graphs showed a poorer prognosis for HER2-enriched and basal subtypes than for both luminal groups (Figure S5). The outcome of HER2-enriched cases was especially worse in the case of OS (Figure S5a). The luminal A has the best survival experience, especially regarding OS and DSS (Figure S5a,b). Cox proportional hazard analysis for all PAM50 subtypes revealed small or neglectable effects for DSS, DFI, and PFI. For OS, we observed the medium effect for HER2-enriched and luminal B subtypes, indicating increased risk compared to basal tumors (Table S7).

Moreover, Tables S8 and S9 show the results of pairwise comparisons between the identified luminal subpopulations for log-rank and Gehan–Wilcoxon, and Cox proportional hazard methods, respectively. Significant differences were observed mainly for comparisons of DFI and PFI of the A3 subpopulation with other luminal tumors. Additionally, we presented the KM graphs for the clusters obtained based on the RNA-seq results [30] in Figure S6. Regardless of the endpoint, both log-rank and Gehan–Wilcoxon tests showed no significant differences in survival between the clusters.

#### 3.1.2. Subpopulation Demographic and Clinical Profile

All identified subpopulations, as well as only luminal ones, differed significantly in age based on the ANOVA test (*p* = 0.0011 and *p* = 0.0231, respectively), although the effect was only small in both cases ($\eta^{2}$ = 0.050 and 0.037, respectively). We obtained similar results for the comparison between all PAM50 subtypes (*p* = 0.0146, $\eta^{2}$ = 0.026). However, for luminal A and B PAM50 subtypes t-test showed no significant age differences (*p* = 0.6334), and Cohen’s *d* effect size was classified as very small.

Table 4 summarizes the relationship between categorical demographic and clinical factors and the luminal subtypes revealed with k-means clustering or given by the PAM50 classifier. We observed a small association between four discovered luminal subpopulations and all categorical factors, apart from ethnicity, metastasis, and hormone therapy, for which the effect was negligible. Nonetheless, for the AJCC node fields and radiotherapy, the Pearson χ^2^ test showed no significant dependency. We noted a similar association for PAM50 subtypes, with negligible effects regarding ethnicity, therapy types, and AJCC node and metastasis fields.

Table S10 shows the results of the dependency analysis for all subtypes proposed here as well as based on the PAM50 predictor, including basal and HER2-enriched. Table S11 summarizes considered demographic and clinical categorical factors.

### 3.2. Molecular Characteristics of Luminal Patient Subpopulations

For 65 proteins out of 166 analyzed (39.16%), we observed at least a large *η^2^* effect size (see Table S12), meaning that the protein abundance differed from other subpopulations for at least one luminal patient subpopulation. In total, 40 of these 65 proteins were previously selected as the most variable by the Gaussian mixture model-based feature selection procedure and were used for patient subpopulation identification by unsupervised clustering methods. Their names are listed in Table S13.

Interestingly, the selected 65 proteins take part in various processes crucial for the proper functioning of the cell. And 25 of those 65 proteins are engaged in the “Pathways in cancer” KEGG pathway (hsa05200), 20 proteins in “PI3K-Akt signaling pathway” (hsa04151), 18 proteins in “Proteoglycans in cancer” (hsa05205), and 17 in “EGFR tyrosine kinase inhibitor resistance” (hsa01521), “Breast cancer” (hsa05224), and “Human cytomegalovirus infection” (hsa05163). Furthermore, selected proteins are abundantly present in Reactome pathways involved in signal transduction, for instance, 30 proteins in “Diseases of signal transduction by growth factor receptors and second messengers” (R-HAS-5663202), 27 proteins in “Cytokine Signaling in Immune system” (R-HAS-1280215), 26 proteins in “Signaling by Receptor Tyrosine Kinases” (R-HAS-9006934), 25 proteins in “Cellular responses to stress” (R-HAS-2262752) and “Cellular responses to stimuli” (R-HAS-8953897), 20 proteins in “PIP3 activates AKT signaling” (R-HSA-1257604), 18 proteins in “PI3K/AKT Signaling in Cancer” (R-HAS-2219528), and 17 proteins in “ESR-mediated signaling” (R-HAS-8939211).

## 4. Discussion

The performed analysis justified dividing the proteomic data set into six subpopulations, with three clusters corresponding to the transcriptomics-based PAM50 luminal A subtype and one cluster per each remaining subtype. This is relatively consistent with the results of similar studies. Hierarchical clustering of RPPA measurements for 403 TCGA-BRCA samples provided seven subgroups [7]. The clusters demonstrated great concordance with PAM50 etiquettes, particularly in terms of basal and HER2-enriched subtypes. Four subgroups corresponded to luminal cases: one mainly to luminal A tumors, one containing both luminal A and B cases, one mostly luminal A contaminated with several luminal B and HER2-enriched cases, and only highly heterogeneous. The size of the seventh cluster did not allow for conclusions. Divisive intelligent K-means (DiviK) [48] clustering of the RNA sequencing data from the TCGA-BRCA project revealed five groups, even though the sample was over two times larger than in this work. Similarly, two of the obtained clusters were highly homogeneous and concordant with PAM50 basal and HER2-enriched subtypes. The remaining ones consisted mainly of luminal tumors: one cluster was almost equally balanced between luminal A and B cases (45.0% and 52.2%, respectively), and two contained mostly luminal A tumors [30].

All the above-mentioned results indicate that distinguishing between luminal A and B tumors remains challenging. Moreover, a part of HER2-enriched cases demonstrates similarities with luminal subtypes. Small and highly heterogeneous subgroups tend to appear among clustering results. Furthermore, the luminal group was reported to be diverse or even a continuum [2].

The proposed statistical testing pipeline allowed us to compare the considered subpopulations of breast cancer patients in terms of their survival. Nevertheless, as indicated by Liu et al. [22] for breast cancer, which is considered to have a relatively good prognosis, the follow-up timespan occurred insufficient to capture enough deaths or recurrences to provide statistical strength in the survival study, in particular for the Cox proportional hazard regression. As mentioned by Liu et al. [22], too short a follow-up period may be especially problematic for ER-positive breast tumors, like luminal ones, recognized as less aggressive and more sensitive to treatment options than ER-negative ones. ER-positive cases account for most of the TCGA-BRCA cohort and, therefore, the sample used for this study. However, those challenges could have been successfully overcome by setting more reliable endpoints like PFI and DFI recommended by Liu et al. [22] and applying dedicated statistical methods, in particular, the Gehan–Wilcoxon test emphasizing early differences in survival outcomes and HR effect size with thresholds adjusted for the group imbalance.

The four newly revealed luminal subpopulations differed in terms of prognosis and survival outcome, which we could not observe when comparing PAM50 transcriptomics-based subtypes luminal A and B. This appears to be a promising advance over the PAM50 classification, in which luminal subtypes did not demonstrate any significant heterogeneity in prognosis, despite better survival outcomes than more aggressive HER2-enriched and basal tumors. Furthermore, no significant differences in survival were also detected in the subset of RNA-seq clustering results.

Luminal A1 and B subpopulations showed similar prognoses, even though they were situated far apart in the UMAP embedding. Out of all proposed subpopulations, the luminal A3 was characterized by the longest survival, especially considering progression- and disease-free intervals. The A2 subpopulation demonstrated the worst prognosis among all luminal ones and, more interestingly, poorer early survival outcome in terms of DFI and PFI than HER2-enriched and basal cases, both considered more aggressive and more likely to develop relapse in the initial years following the diagnosis and treatment. On the other hand, the DSS for the luminal A2 subpopulation was much better.

The association between proposed subpopulations and demographic or clinical categorical factors, in general, remained minor and comparable to that observed for the PAM50 classification. Although patient age at diagnosis differed significantly among both all subpopulations and the luminal subset, the effect size was small. The differentiation was similar for subpopulations identified based on proteomic data and PAM50 subtypes. Hence, the obtained groups, including additional luminal ones, did not reflect the cancer stage or patient demographic background more than PAM50 subtypes. Therefore, we suspect the molecular background and tumor biology underlie the heterogeneity revealed by clustering protein measurements rather than demographic or clinical factors. Further investigation is needed to characterize the molecular portraits of the identified subpopulations.

Various proteins with well-recognized roles in breast cancer, including estrogen and progesterone receptors or GATA3 transcription factor, were differentially expressed between the identified four luminal subpopulations. The luminal A2 overexpressed ER and PR, while luminal A1 had their lowest levels. GATA3, which was underexpressed in the luminal A1 subpopulation, can serve as a diagnostic marker for luminal A and B subtypes, creating a transcription factors’ network with ER and FOXA1 [49,50]. Caveolin, overexpressed in luminal A1 and A3 subpopulations, was reported to both suppress and promote breast cancer progression [51,52,53]. The RB1 protein, with elevated levels in luminal A1 cases, was associated with therapy response mainly in TNBCs [54], and the loss of heterozygosity has been observed at its locus in basal and luminal B tumors [55]. AKT protein, with lower levels in luminal B tumors, plays a crucial role in the PI3K-AKT signaling pathway in cell metabolism, growth, proliferation, apoptosis, angiogenesis, and carcinogenesis [56,57,58]. RBM15, regulated by BARX2 and ER, has been reported to affect cell growth and invasion in breast cancer samples [59,60].

To summarize, the subpopulations demonstrate considerable diversity in survival outcomes and remain only negligibly affected or biased by demographic factors. We did not discover a strong impact of clinical factors like cancer stage on the proposed subpopulations.

## 5. Conclusions

The proposed subtyping based on the proteomic profile complemented the well-established intrinsic molecular classification of breast cancer with additional information on heterogeneity undetected by the gene expression profiling. Proteomic-based patient subpopulations, including four luminal ones instead of only two currently recognized subtypes, varied in the prognosis to the extent not observed in PAM50 transcriptomics-based classification. The differences in survival outcomes were especially noted regarding the time to new cancer events. One of the novel luminal subpopulations (luminal A2) demonstrated a prognosis poorer than HER2-enriched and basal tumors. The other one (luminal A3) had the best survival outcome. Moreover, the identified subpopulations showed a minor association with demographic and clinical factors, comparable to well-established PAM50-based subtypes. However, unlike the PAM50 luminal A and B subtypes, the novel luminal subpopulations demonstrated a small dependency on the lymph nodes affected.

Therefore, protein level profiling may deliver a more comprehensive insight into tumor biology and provide clinically relevant information. Nonetheless, further investigation is required for a better understanding of the molecular differences between the revealed subpopulations and for examining the potential prognostic or clinical applications of the presented findings.

## Figures and Tables

**Table 1:** Number and percentage of patients in proteomics-based subpopulations referred to PAM50 subtypes.

PAM50 Subtype	Proteomics-Based Subpopulation						TOTAL
	Basal	HER2-Enriched	Luminal				
			A1	A2	A3	B	
Basal	79 (88.76%)	0 (0.00%)	4 (9.09%)	0 (0.00%)	2 (2.30%)	1 (1.39%)	86 (21.13%)
HER2-enriched	8 (8.99%)	34 (62.96%)	2 (4.55%)	0 (0.00%)	2 (2.30%)	4 (5.56%)	50 (12.29%)
Luminal A	2 (2.25%)	9 (16.67%)	27 (61.36%)	47 (77.05%)	65 (74.71%)	23 (31.94%)	173 (42.51%)
Luminal B	0 (0.00%)	11 (20.37%)	11 (25.00%)	14 (22.95%)	18 (20.69%)	44 (61.11%)	98 (24.08%)
TOTAL	89 (100.00%)	54 (100.00%)	44 (100.00%)	61 (100.00%)	87 (100.00%)	72 (100.00%)	407 (100.00%)

**Table 2:** Cox proportional hazard analysis of identified luminal subpopulations.

Subpopulation	N	N_e_	N_c_	HR	HR Effect	HR Allocation Adjusted Critical Value		
						α = 0.1 Small Effect	α = 0.3 Medium Effect	α = 0.5 Large Effect
Overall Survival								
Luminal A1	44	8	36	0.657	Small	HR < 0.773; HR > 1.293	HR < 0.47; HR > 2.13	HR < 0.275; HR > 3.636
Luminal A2	61	14	47	1.275	Small	HR < 0.805; HR > 1.242	HR < 0.517; HR > 1.934	HR < 0.314; HR > 3.18
Luminal A3	87	9	78	0.533	Medium	HR < 0.831; HR > 1.203	HR < 0.561; HR > 1.783	HR < 0.354; HR > 2.828
Luminal B	72	9	63	Reference				
Disease-Specific Survival								
Luminal A1	43	5	38	0.759	Small	HR < 0.771; HR > 1.297	HR < 0.466; HR > 2.146	HR < 0.272; HR > 3.674
Luminal A2	58	4	54	0.776	Small	HR < 0.801; HR > 1.249	HR < 0.51; HR > 1.961	HR < 0.309; HR > 3.241
Luminal A3	85	2	83	0.213	Large	HR < 0.83; HR > 1.205	HR < 0.558; HR > 1.792	HR < 0.351; HR > 2.847
Luminal B	72	5	67	Reference				
Disease-Free Interval								
Luminal A1	38	4	34	0.927	No effect	HR < 0.77; HR > 1.298	HR < 0.465; HR > 2.15	HR < 0.271; HR > 3.684
Luminal A2	46	6	40	1.748	Small	HR < 0.79; HR > 1.266	HR < 0.494; HR > 2.025	HR < 0.295; HR > 3.391
Luminal A3	79	2	77	0.250	Large	HR < 0.833; HR > 1.201	HR < 0.563; HR > 1.776	HR < 0.356; HR > 2.81
Luminal B	64	5	59	Reference				
Progression-Free Interval								
Luminal A1	44	7	37	0.839	No effect	HR < 0.773; HR > 1.293	HR < 0.47; HR > 2.13	HR < 0.275; HR > 3.636
Luminal A2	61	9	52	1.101	No effect	HR < 0.805; HR > 1.242	HR < 0.517; HR > 1.934	HR < 0.314; HR > 3.18
Luminal A3	87	5	82	0.359	Medium	HR < 0.831; HR > 1.203	HR < 0.561; HR > 1.783	HR < 0.354; HR > 2.828
Luminal B	72	8	64	Reference				

**Table 3:** Cox proportional hazard analysis of luminal PAM50 subtypes.

PAM50 Subtype	N	N_e_	N_c_	HR	HR Effect	HR Allocation Adjusted Critical Value		
						α = 0.1 Small Effect	α = 0.3 Medium Effect	α = 0.5 Large Effect
Overall Survival								
Luminal A	173	26	147	0.612	Small	HR < 0.852; HR > 1.174	HR < 0.598; HR > 1.671	HR < 0.39; HR > 2.566
Luminal B	98	16	82	Reference				
Disease-Specific Survival								
Luminal A	169	10	159	0.437	Medium	HR < 0.852; HR > 1.174	HR < 0.598; HR > 1.672	HR < 0.389; HR > 2.568
Luminal B	96	8	88	Reference				
Disease-Free Interval								
Luminal A	147	11	136	1.046	No effect	HR < 0.852; HR > 1.173	HR < 0.6; HR > 1.668	HR < 0.391; HR > 2.558
Luminal B	82	5	77	Reference				
Progression-Free Interval								
Luminal A	173	20	153	0.752	Small	HR < 0.852; HR > 1.174	HR < 0.598; HR > 1.671	HR < 0.39; HR > 2.566
Luminal B	98	11	87	Reference				

**Table 4:** Association between categorical demographic and clinical factors and luminal subtypes identified with k-means clustering of protein levels or based on PAM50 classifier.

Feature	χ^2^	*p*-Value	Cramér’s V	Cramér’s V Effect Threshold		
				Small	Medium	Large
Proteomics-based subpopulations						
Race	13.42	0.0368	0.1712	0.0707	0.2121	0.3536
Ethnicity	0.23	0.9718	0.0346	0.1	0.3	0.5
AJCC Stage	18.61	0.0287	0.1536	0.0577	0.1732	0.2887
AJCC Tumor	19.34	0.0225	0.1566			
AJCC Node	13.23	0.1526	0.1292			
AJCC Tumor Binarized	13.86	0.0031	0.2295	0.1	0.3	0.5
AJCC Node Binarized	3.75	0.2900	0.1191			
AJCC Metastasis	2.23	0.5254	0.0922			
Radiotherapy	4.42	0.2193	0.1294			
Chemotherapy	12.37	0.0062	0.2165			
Hormone Therapy	2.11	0.5500	0.0894			
PAM50-based subtypes						
Race	3.74	0.1543	0.1269	0.1	0.3	0.5
Ethnicity	1.26	0.2610	0.0793			
AJCC Stage	9.19	0.0269	0.1848			
AJCC Tumor	14.40	0.0024	0.2309			
AJCC Node	0.91	0.8228	0.0580			
AJCC Tumor Binarized	13.25	0.0003	0.2215			
AJCC Node Binarized	0.67	0.4133	0.0497			
AJCC Metastasis	1.42	0.2335	0.0725			
Radiotherapy	0.05	0.8295	0.0131			
Chemotherapy	1.45	0.2280	0.0732			
Hormone Therapy	0.09	0.7613	0.0185			

Table cells with Cramér’s V values are colored based on the effect size interpretation. Cramér’s V effect interpretation thresholds were adjusted for the size of corresponding contingency tables.

## Data Availability

The results shown here are based upon data generated by the TCGA Research Network: https://www.cancer.gov/tcga (accessed on 2 February 2021).

## Supplementary Materials

The following supporting information can be downloaded at https://www.mdpi.com/article/10.3390/cancers15174230/s1. Table S1: The list of 86 proteins selected with the Gaussian-mixture-model-based feature filtration; Figure S1: The overlapping of patients between the data sets of all available PAM50 labels, protein levels measured with Reverse Phase Protein Arrays (RPPA), and RNA sequencing measurements used by Tobiasz et al.; Table S2: Number and percentage of patients in proteomics-based subpopulations referred to clusters obtained based on the RNA sequencing results; Figure S2: Proteomics-based subpopulations related to clusters obtained based on the RNA sequencing results; Table S3: Number and percentage of patients in clusters obtained based on the RNA sequencing results referred to PAM50 subtypes; Figure S3: Protein-level-based UMAP visualization with data points colored with regard to breast cancer subtypes. (a) Breast cancer subtypes obtained with the PAM50 transcriptomic-based predictor. (b) Breast cancer patients’ subtypes resulting from clustering of RNA sequencing results; Figure S4: Kaplan–Meier survival curves of all subpopulations identified with k-means clustering of protein levels. (a) Overall Survival; (b) Disease-Specific Survival; (c) Disease-Free Interval; (d) Progression-Free Interval; Figure S5: Kaplan–Meier survival curves of all PAM50 subtypes. (a) Overall Survival; (b) Disease-Specific Survival; (c) Disease-Free Interval; (d) Progression-Free Interval; Table S4: Results of log-rank and Gehan–Wilcoxon tests for luminal subtypes identified with k-means clustering or PAM50 classifier; Table S5: Results of log-rank and Gehan–Wilcoxon tests for all subtypes identified with k-means clustering or PAM50 classifier; Table S6: Cox proportional hazard analysis of all identified subpopulations; Table S7: Cox proportional hazard analysis of all PAM50 subtypes; Table S8: Results of log-rank and Gehan–Wilcoxon tests for pairwise comparisons of luminal subpopulations identified with k-means clustering; Table S9: Cox proportional hazard pairwise analysis of luminal subpopulations identified with k-means clustering; Figure S6: Kaplan–Meier survival curves of the clusters obtained based on the RNA sequencing results. (a) Overall Survival; (b) Disease-Specific Survival; (c) Disease-Free Interval; (d) Progression-Free Interval; Table S10: Association between categorical demographic and clinical factors and all subtypes identified with k-means clustering or based on PAM50 classifier; Table S11: Summary of demographic and clinical categorical data; Table S12: The list of proteins with at least a large *η^2^* effect between the identified four luminal subpopulations; Table S13: The list of 40 proteins selected with the Gaussian-mixture-model-based feature filtration and with at least a large *η^2^* effect between the identified four luminal subpopulations.
