# Supplementary material for: Proteomic Profile Distinguishes New Subpopulations of Breast Cancer Patients with Different Survival Outcomes
Source: Cancers (Basel). 2023 Aug 24;15(17):4230. doi: 10.3390/cancers15174230 (PMC10486506; doi:10.3390/cancers15174230)
Supplement: Supplementary file 1 [file cancers-15-04230-s001.zip › cancers-2550928-supplementary.pdf]

Proteomic Profile Distinguishes New Subpopulations of Breast Cancer Patients with Different Survival Outcomes

Joanna Tobiasz [1,2] and Joanna Polanska [1,*]

[1] Department of Data Science and Engineering, Silesian University of Technology, 44-100 Gliwice, Poland; joanna.tobiasz@polsl.pl
[2] Department of Computer Graphics, Vision and Digital Systems, Silesian University of Technology, 44-100 Gliwice, Poland
* Correspondence: joanna.polanska@polsl.pl

Supplementary Materials

Table S1. The list of 86 proteins selected with the Gaussian-mixture-model-based feature filtration.

Selected proteins
14-3-3_zeta-R-V
4E-BP1-R-V
4E-BP1_pT37_T46-R-V
ACC_pS79-R-V
ADAR1-M-V
Akt-R-V
Akt_pS473-R-V
Akt_pT308-R-V
AMPK_pT172-R-V
Annexin-1-M-E
AR-R-V
ASNS-R-V
Bcl-2-M-V
Bcl2A1-R-V
beta-Catenin-R-V
Bim-R-V
B-Raf_pS445-R-V
BRD4-R-V
Caspase-7_cleavedD198-R-C
Caveolin-1-R-V
CD31-M-V
CDK1_pY15-R-C
c-Kit-R-V
Claudin-7-R-V
c-Myc-R-C
COG3-R-V
Collagen_VI-R-V

Selected proteins
Cyclin_B1-R-V
Cyclin_D1-R-V
Cyclin_E1-M-V
DUSP4-R-V
E-Cadherin-R-V
eEF2K-R-V
EGFR_pY1068-R-C
eIF4G-R-C
ER-alpha-R-V
ER-alpha_pS118-R-V
ERK2-R-E
FASN-R-V
Fibronectin-R-V
FoxM1-R-V
G6PD-M-V
GAB2-R-V
GAPDH-M-C
GATA3-M-V
GSK3-alpha-beta-M-V
GSK3-alpha-beta_pS21_S9-R-V
GSK3_pS9-R-V
HER2-M-V
HSP70-R-C
IGFBP2-R-V
Ku80-R-C
Lck-R-V
MAPK_pT202_Y204-R-V
MEK1-R-V
mTOR-R-V
MYH11-R-V
Myosin-IIa_pS1943-R-V
NDRG1_pT346-R-V
NF-kB-p65_pS536-R-C
p16_INK4a-R-V
p27-R-V
p38_pT180_Y182-R-V
p70S6K-R-V
PDCD4-R-C
PKC-pan_BetaII_pS660-R-V
PR-R-V
PREX1-R-E
PTEN-R-V
Rab25-R-V
RBM15-R-V

Selected proteins
Rb_pS807_S811-R-V
Rictor-R-C
S6-R-E
S6_pS235_S236-R-V
S6_pS240_S244-R-V
SCD-M-V
SHP-2_pY542-R-C
Smac-M-E
Src_pY416-R-C
Src_pY527-R-V
STAT3_pY705-R-V
STAT5-alpha-R-V
Syk-M-V
TFRC-R-V
TSC1-R-C

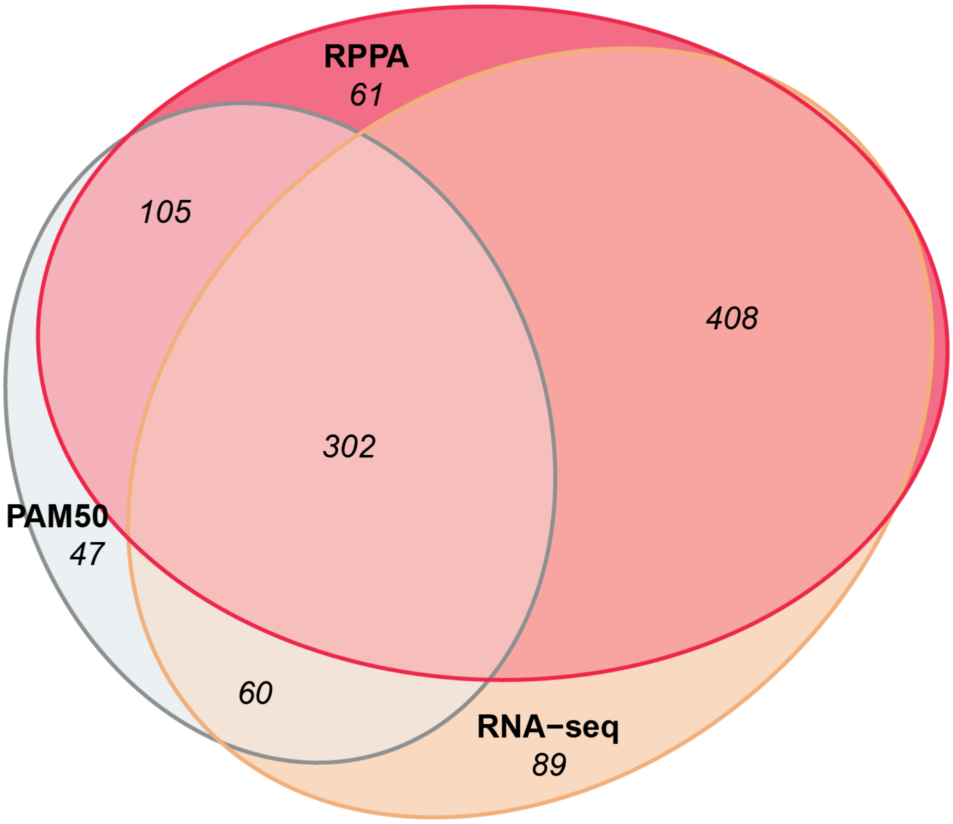

Figure S1. The overlapping of patients between the data sets of all available PAM50 labels, protein levels measured with Reverse Phase Protein Arrays (RPPA), and RNA sequencing measurements used by Tobiasz et al. [30].

Table S2. Number and percentage of patients in proteomics-based subpopulations referred to clusters obtained based on the RNA sequencing results [30].

RNA-seq-based cluster	Proteomics-based subpopulation						TOTAL
	Basal	HER2-enriched	Luminal				
			A1	A2	A3	B	
1	**0** *(0.00%)*	**3** *(9.38%)*	**5** *(13.51%)*	**21** *(50.00%)*	**11** *(16.42%)*	**12** *(22.22%)*	**52** *(17.22%)*
2	**63** *(90.00%)*	**0** *(0.00%)*	**3** *(8.11%)*	**0** *(0.00%)*	**0** *(0.00%)*	**0** *(0.00%)*	**66** *(21.85%)*
3	**7** *(10.00%)*	**22** *(68.75%)*	**3** *(8.11%)*	**0** *(0.00%)*	**4** *(5.97%)*	**4** *(7.41%)*	**40** *(13.25%)*
4	**0** *(0.00%)*	**2** *(6.25%)*	**6** *(16.22%)*	**5** *(11.90%)*	**13** *(19.40%)*	**31** *(57.41%)*	**57** *(18.87%)*
5	**0** *(0.00%)*	**5** *(15.62%)*	**20** *(54.05%)*	**16** *(38.10%)*	**39** *(58.21%)*	**7** *(12.96%)*	**87** *(28.81%)*
TOTAL	**70** *(100.00%)*	**32** *(100.00%)*	**37** *(100.00%)*	**42** *(100.00%)*	**67** *(100.00%)*	**54** *(100.00%)*	**302** *(100.00%)*

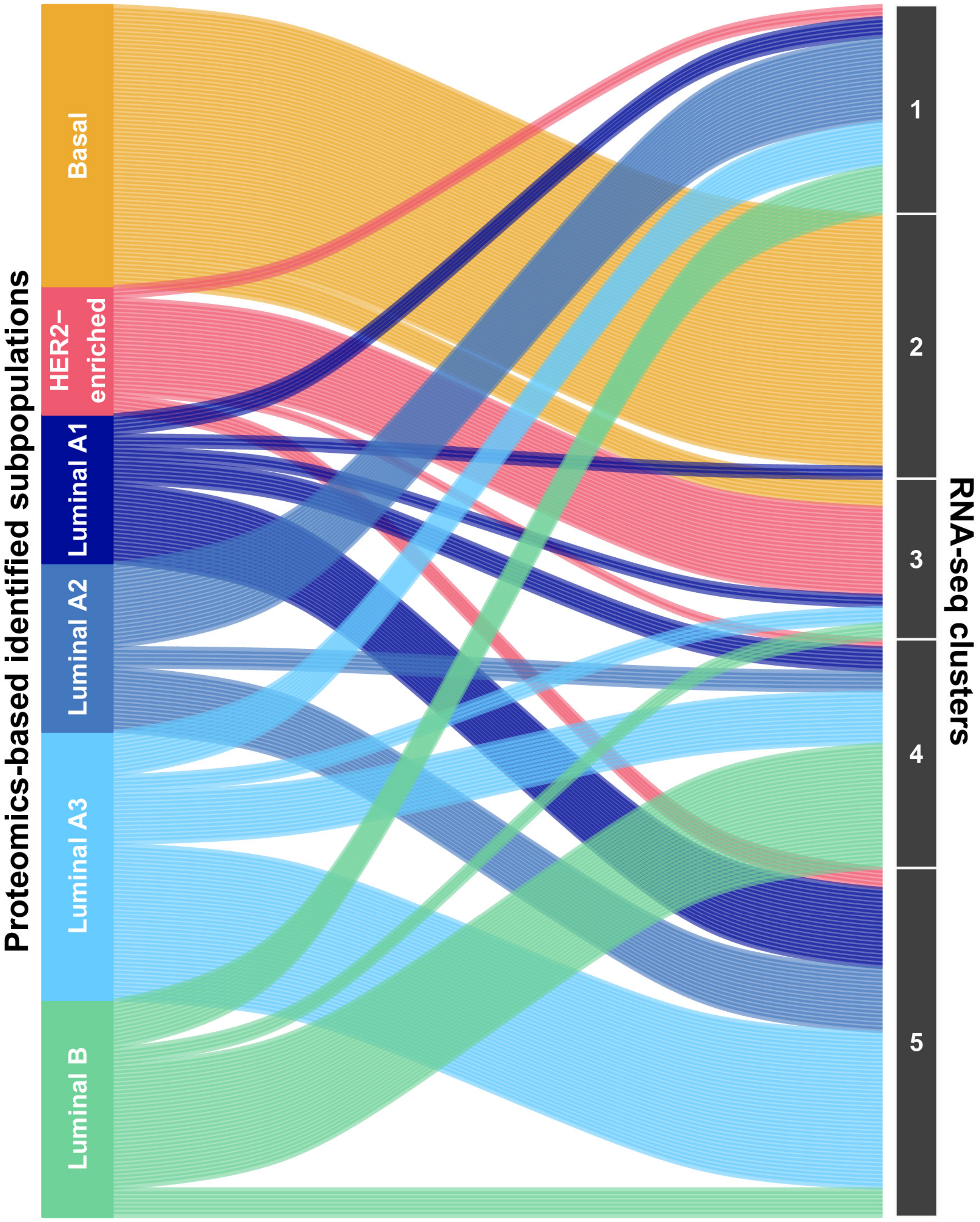

Figure S2. Proteomics-based subpopulations related to clusters obtained based on the RNA sequencing results [30].

Table S3. Number and percentage of patients in clusters obtained based on the RNA sequencing results [30] referred to PAM50 subtypes.

PAM50 subtype	RNA-seq-based cluster				
	**1**	**2**	**3**	**4**	**5**
**Basal**	**1** *(1.92%)*	**65** *(98.48%)*	**2** *(5.00%)*	**1** *(1.75%)*	**1** *(1.15%)*
**HER2-enriched**	**4** *(7.69%)*	**0** *(0.00%)*	**24** *(60.00%)*	**0** *(0.00%)*	**3** *(3.45%)*
**Luminal A**	**32** *(61.54%)*	**1** *(1.52%)*	**6** *(15.00%)*	**24** *(42.11%)*	**74** *(85.06%)*
**Luminal B**	**15** *(28.85%)*	**0** *(0.00%)*	**8** *(20.00%)*	**32** *(56.14%)*	**9** *(10.34%)*
**TOTAL**	**52** *(100.00%)*	**66** *(100.00%)*	**40** *(100.00%)*	**57** *(100.00%)*	**87** *(100.00%)*

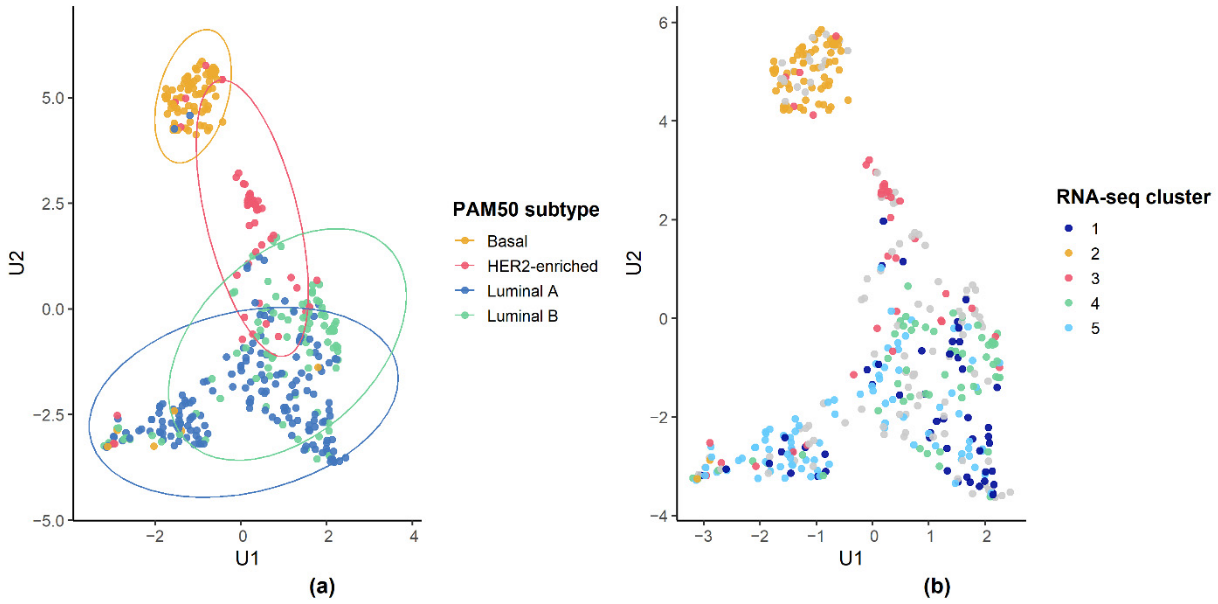

Figure S3. Protein-level-based UMAP visualization with data points colored with regard to breast cancer subtypes. (**a**) Breast cancer subtypes obtained with the PAM50 transcriptomic-based predictor. (**b**) Breast cancer patients' subtypes resulting from clustering of RNA sequencing results [30].

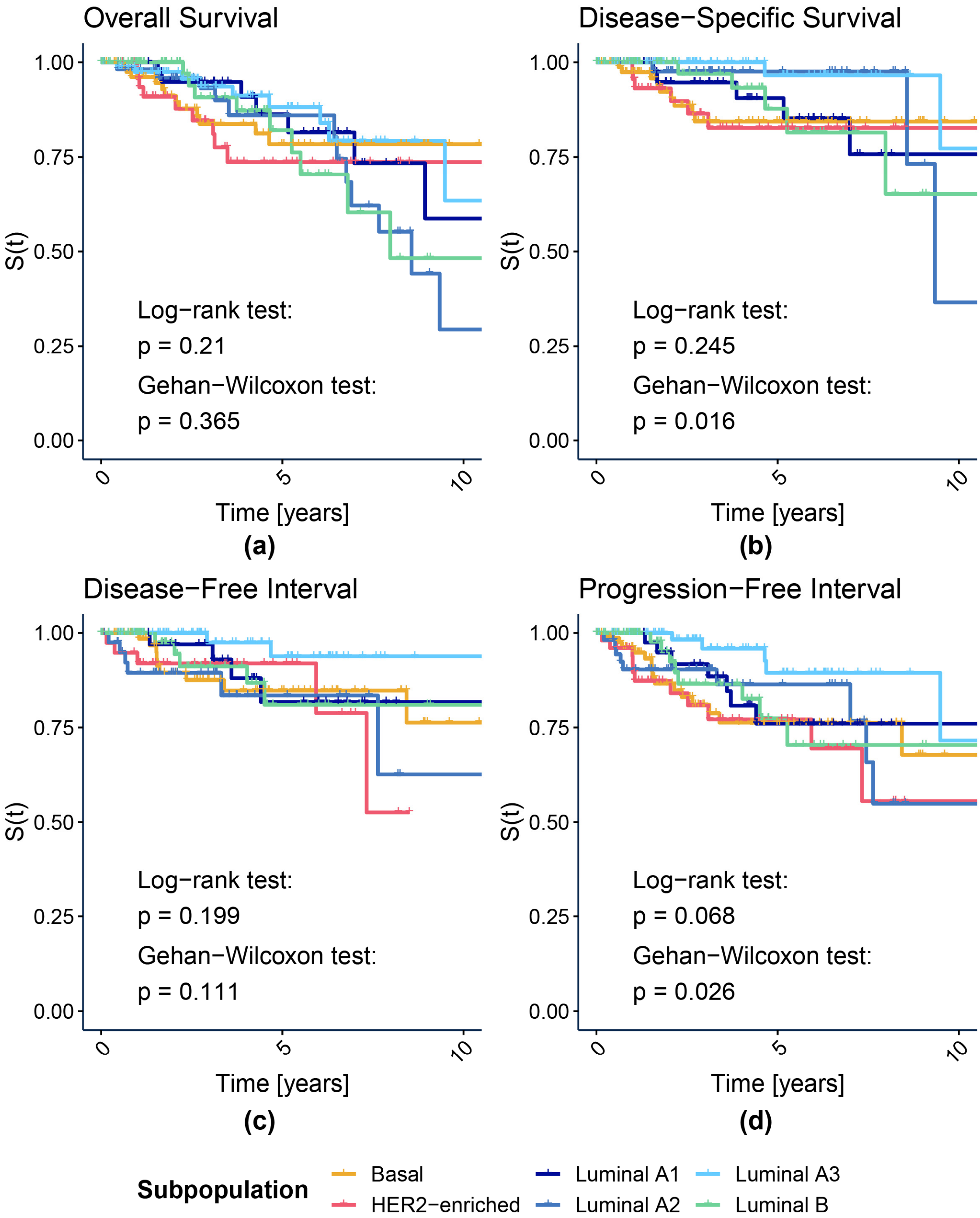

Figure S4. Kaplan-Meier survival curves of all subpopulations identified with k-means clustering of protein levels. (**a**) Overall Survival; (**b**) Disease-Specific Survival; (**c**) Disease-Free Interval; (**d**) Progression-Free Interval.

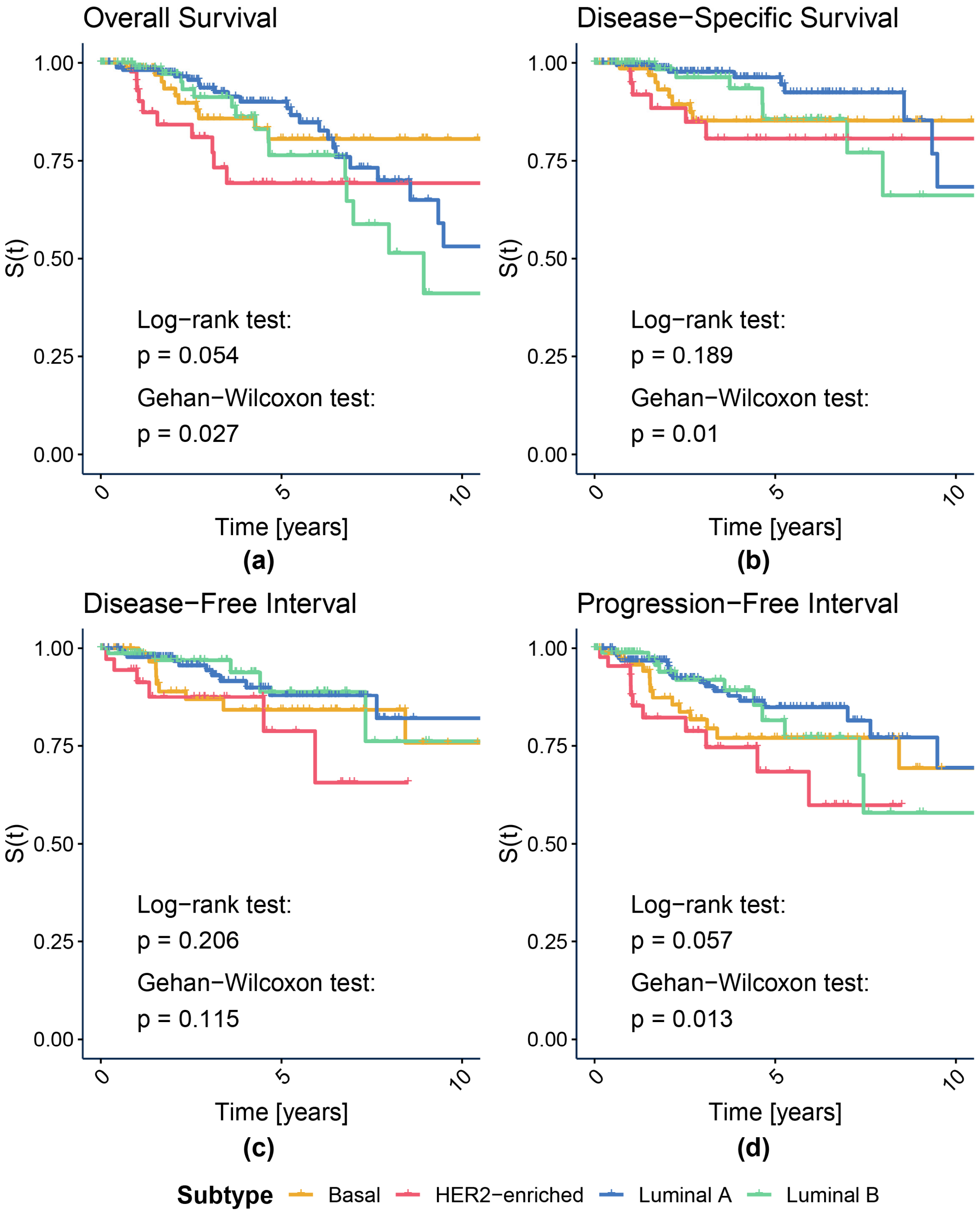

Figure S5. Kaplan-Meier survival curves of all PAM50 subtypes. (**a**) Overall Survival; (**b**) Disease-Specific Survival; (**c**) Disease-Free Interval; (**d**) Progression-Free Interval.

Table S4. Results of log-rank and Gehan-Wilcoxon tests for luminal subtypes identified with k-means clustering or PAM50 classifier.

Endpoint type	χ^2		p-value	
	Log-rank test	Gehan-Wilcoxon test	Log-rank test	Gehan-Wilcoxon test
**Proteomics-based subpopulations**				
**Overall Survival**	4.99	0.68	0.1724	0.8788
**Disease-Specific Survival**	4.06	5.08	0.2552	0.1661
**Disease-Free Interval**	6.97	9.12	0.0730	0.0277
**Progression-Free Interval**	4.87	6.41	0.1818	0.0932
**PAM50-based subtypes**				
**Overall Survival**	2.32	0.57	0.1280	0.4521
**Disease-Specific Survival**	3.01	0.70	0.0828	0.4043
**Disease-Free Interval**	0.01	0.10	0.9333	0.7488
**Progression-Free Interval**	0.56	0.003	0.4530	0.9512

Table S5. Results of log-rank and Gehan-Wilcoxon tests for all subtypes identified with k-means clustering or PAM50 classifier.

Endpoint type	χ^2		p-value	
	Log-rank test	Gehan-Wilcoxon test	Log-rank test	Gehan-Wilcoxon test
**Proteomics-based subpopulations**				
**Overall Survival**	:148	8177	3153<<	31697;
**Disease-Specific Survival**	919<	461<7	31577:	313493
**Disease-Free Interval**	:164	;1<9	314<;9	31443;
**Progression-Free Interval**	4315;	451:3	3139::	313597
**PAM50-based subtypes**				
**Overall Survival**	:199	<14;	313869	3135:3
**Disease-Specific Survival**	71:;	4415:	314;;;	313436
**Disease-Free Interval**	718:	81<6	315393	314486
**Progression-Free Interval**	:185	431:5	3138:4	313466

Table S6. Cox proportional hazard analysis of all identified subpopulations.

Subpopulation	N	N_e	N_c	HR	HR effect	HR allocation adjusted critical value		
						α = 0.1 small effect	α = 0.3 medium effect	α = 0.5 large effect
**Overall Survival**								
**Basal**	89	13	76	Reference				
**HER2-enriched**	54	11	43	1.836	Small	HR < 0.773; HR > 1.294	HR < 0.468; HR > 2.135	HR < 0.274; HR > 3.648
**Luminal A1**	44	8	36	1.050	No effect	HR < 0.749; HR > 1.336	HR < 0.436; HR > 2.295	HR < 0.249; HR > 4.023
**Luminal A2**	61	14	47	2.016	Small	HR < 0.785; HR > 1.273	HR < 0.487; HR > 2.054	HR < 0.289; HR > 3.459
**Luminal A3**	87	9	78	0.837	No effect	HR < 0.816; HR > 1.225	HR < 0.536; HR > 1.867	HR < 0.331; HR > 3.023
**Luminal B**	72	9	63	1.445	Small	HR < 0.801; HR > 1.248	HR < 0.511; HR > 1.958	HR < 0.309; HR > 3.236
**Disease-Specific Survival**								
**Basal**	87	9	78	Reference				
**HER2-enriched**	52	7	45	1.579	Small	HR < 0.771; HR > 1.297	HR < 0.466; HR > 2.146	HR < 0.272; HR > 3.673
**Luminal A1**	43	5	38	0.957	No effect	HR < 0.749; HR > 1.336	HR < 0.436; HR > 2.296	HR < 0.249; HR > 4.023
**Luminal A2**	58	4	54	0.817	No effect	HR < 0.783; HR > 1.278	HR < 0.483; HR > 2.071	HR < 0.286; HR > 3.5
**Luminal A3**	85	2	83	0.241	Large	HR < 0.816; HR > 1.225	HR < 0.536; HR > 1.867	HR < 0.331; HR > 3.024
**Luminal B**	72	5	67	1.002	No effect	HR < 0.803; HR > 1.245	HR < 0.514; HR > 1.946	HR < 0.312; HR > 3.208
**Disease-Free Interval**								
**Basal**	79	9	70	Reference				
**HER2-enriched**	41	5	36	1.315	No effect	HR < 0.755; HR > 1.325	HR < 0.444; HR > 2.254	HR < 0.255; HR > 3.927
**Luminal A1**	38	4	34	0.819	No effect	HR < 0.745; HR > 1.342	HR < 0.431; HR > 2.32	HR < 0.245; HR > 4.079
**Luminal A2**	46	6	40	1.516	Small	HR < 0.768; HR > 1.302	HR < 0.462; HR > 2.165	HR < 0.269; HR > 3.717
**Luminal A3**	79	2	77	0.225	Large	HR < 0.818; HR > 1.222	HR < 0.538; HR > 1.857	HR < 0.333; HR > 3
**Luminal B**	64	5	59	0.884	No effect	HR < 0.801; HR > 1.248	HR < 0.511; HR > 1.958	HR < 0.309; HR > 3.234
**Progression-Free Interval**								
**Basal**	89	15	74	Reference				
**HER2-enriched**	54	12	42	1.483	Small	HR < 0.773; HR > 1.294	HR < 0.468; HR > 2.135	HR < 0.274; HR > 3.648
**Luminal A1**	44	7	37	0.738	Small	HR < 0.749; HR > 1.336	HR < 0.436; HR > 2.295	HR < 0.249; HR > 4.023

Subpo-pulation	N	N_e	N_c	HR	HR effect	HR allocation adjusted critical value		
						α = 0.1 small effect	α = 0.3 medium effect	α = 0.5 large effect
**Luminal A2**	61	9	52	0.965	No effect	HR < 0.785; HR > 1.273	HR < 0.487; HR > 2.054	HR < 0.289; HR > 3.459
**Luminal A3**	87	5	82	0.308	Large	HR < 0.816; HR > 1.225	HR < 0.536; HR > 1.867	HR < 0.331; HR > 3.023
**Luminal B**	72	8	64	0.846	No effect	HR < 0.801; HR > 1.248	HR < 0.511; HR > 1.958	HR < 0.309; HR > 3.236

Table S7. Cox proportional hazard analysis of all PAM50 subtypes.

PAM50 subtype	N	N_e	N_c	HR	HR effect	HR allocation adjusted critical value		
						α = 0.1 small effect	α = 0.3 medium effect	α = 0.5 large effect
**Overall Survival**								
**Basal**	86	11	75	Reference				
**HER2**	50	11	39	2.830	Medium	HR < 0.768; HR > 1.302	HR < 0.462; HR > 2.166	HR < 0.269; HR > 3.72
**LumA**	173	26	147	1.408	Small	HR < 0.857; HR > 1.166	HR < 0.609; HR > 1.642	HR < 0.4; HR > 2.497
**LumB**	98	16	82	2.137	Medium	HR < 0.827; HR > 1.209	HR < 0.554; HR > 1.805	HR < 0.348; HR > 2.878
**Disease-Specific Survival**								
**Basal**	84	8	76	Reference				
**HER2**	48	6	42	1.977	Small	HR < 0.766; HR > 1.306	HR < 0.459; HR > 2.179	HR < 0.267; HR > 3.75
**LumA**	169	10	159	0.691	Small	HR < 0.857; HR > 1.166	HR < 0.609; HR > 1.642	HR < 0.4; HR > 2.497
**LumB**	96	8	88	1.326	Small	HR < 0.828; HR > 1.208	HR < 0.554; HR > 1.804	HR < 0.348; HR > 2.875
**Disease-Free Interval**								
**Basal**	76	9	67	Reference				
**HER2**	42	6	36	1.723	Small	HR < 0.762; HR > 1.312	HR < 0.454; HR > 2.204	HR < 0.262; HR > 3.81
**LumA**	147	11	136	0.664	Small	HR < 0.856; HR > 1.169	HR < 0.606; HR > 1.65	HR < 0.397; HR > 2.517
**LumB**	82	5	77	0.624	Small	HR < 0.824; HR > 1.214	HR < 0.548; HR > 1.826	HR < 0.342; HR > 2.927
**Progression-Free Interval**								
**Basal**	86	14	72	Reference				
**HER2**	50	11	39	1.840	Small	HR < 0.768; HR > 1.302	HR < 0.462; HR > 2.166	HR < 0.269; HR > 3.72
**LumA**	173	20	153	0.679	Small	HR < 0.857; HR > 1.166	HR < 0.609; HR > 1.642	HR < 0.4; HR > 2.497
**LumB**	98	11	87	0.858	No effect	HR < 0.827;	HR < 0.554;	HR < 0.348;

PAM50 subtype	N	N_e	N_c	HR	HR effect	HR allocation adjusted critical value		
						$\alpha = 0.1$ small effect	$\alpha = 0.3$ medium effect	$\alpha = 0.5$ large effect
						HR > 1.209	HR > 1.805	HR > 2.878

"HER2" denotes HER2-enriched subtype, "LumA" luminal A subtype, and "LumB" luminal B subtype.

Table S8. Results of log-rank and Gehan-Wilcoxon tests for pairwise comparisons of luminal subpopulations identified with k-means clustering.

Comparison	χ^2		p-value	
	Log-rank test	Gehan-Wilcoxon test	Log-rank test	Gehan-Wilcoxon test
**Overall Survival**				
**A1 versus A3**	0.37	0.04	0.5432	0.8430
**A2 versus A3**	3.80	0.43	0.0511	0.5138
**B versus A3**	1.38	0.10	0.2395	0.7578
**A2 versus A1**	2.93	0.61	0.0872	0.4358
**B versus A1**	0.54	0.10	0.4629	0.7518
**A2 versus B**	0.15	0.09	0.7020	0.7632
**Disease-Specific Survival**				
**A1 versus A3**	2.83	4.70	0.0925	0.0302
**A2 versus A3**	4.04	1.23	0.0444	0.2670
**B versus A3**	3.84	4.10	0.0499	0.0428
**A2 versus A1**	0.00	0.93	0.9867	0.3337
**B versus A1**	0.00	0.25	0.9812	0.6187
**A2 versus B**	0.23	0.43	0.6334	0.5104
**Disease-Free Interval**				
**A1 versus A3**	2.59	3.02	0.1074	0.0824
**A2 versus A3**	6.74	7.52	0.0094	0.0061
**B versus A3**	3.59	4.35	0.0583	0.0370
**A2 versus A1**	0.85	1.34	0.3567	0.2474
**B versus A1**	0.02	0.04	0.8937	0.8359
**A2 versus B**	0.86	2.18	0.3525	0.1398
**Progression-Free Interval**				
**A1 versus A3**	2.28	4.18	0.1313	0.0409
**A2 versus A3**	4.30	5.41	0.0382	0.0201
**B versus A3**	4.24	5.36	0.0394	0.0207
**A2 versus A1**	0.17	0.22	0.6803	0.6363
**B versus A1**	0.04	0.01	0.8333	0.9235
**A2 versus B**	0.01	0.62	0.9033	0.4297

Table S9. Cox proportional hazard pairwise analysis of luminal subpopulations identified with k-means clustering.

Comparison	Subpopulation	N	N_e	N_c	HR	HR effect	HR allocation adjusted critical value		
							$\alpha = 0.1$	$\alpha = 0.3$	$\alpha = 0.5$
							small effect	medium effect	large effect
**Overall Survival**									
A1 vs A3	A1	44	8	36	1.344	Small	HR < 0.751; HR > 1.331	HR < 0.439; HR > 2.276	HR < 0.251; HR > 3.977
	A3	87	9	78	Reference				
A2 vs A3	A2	61	14	47	2.278	Medium	HR < 0.788; HR > 1.27	HR < 0.49; HR > 2.04	HR < 0.292; HR > 3.426
	A3	87	9	78	Reference				
B vs A3	A3	87	9	78	Reference				
	B	72	9	63	1.740	Small	HR < 0.803; HR > 1.245	HR < 0.514; HR > 1.946	HR < 0.312; HR > 3.208
A2 vs A1	A1	44	8	36	Reference				
	A2	61	14	47	2.196	Medium	HR < 0.839; HR > 1.191	HR < 0.575; HR > 1.738	HR < 0.367; HR > 2.721
B vs A1	A1	44	8	36	Reference				
	B	72	9	63	1.448	Small	HR < 0.848; HR > 1.179	HR < 0.592; HR > 1.69	HR < 0.383; HR > 2.611
A2 vs B	A2	61	14	47	1.180	No effect	HR < 0.805; HR > 1.242	HR < 0.517; HR > 1.934	HR < 0.314; HR > 3.18
	B	72	9	63	Reference				
**Disease-Specific Survival**									
A1 vs A3	A1	43	5	38	3.725	Medium	HR < 0.751; HR > 1.331	HR < 0.439; HR > 2.276	HR < 0.251; HR > 3.977
	A3	85	2	83	Reference				
A2 vs A3	A2	58	4	54	5.078	Large	HR < 0.785; HR > 1.274	HR < 0.486; HR > 2.057	HR < 0.289; HR > 3.466
	A3	85	2	83	Reference				
B vs A3	A3	85	2	83	Reference				
	B	72	5	67	4.668	Large	HR < 0.805; HR > 1.242	HR < 0.517; HR > 1.935	HR < 0.314; HR > 3.181
A2 vs A1	A1	43	5	38	Reference				
	A2	58	4	54	1.011	No effect	HR < 0.838; HR > 1.193	HR < 0.573; HR > 1.746	HR < 0.365; HR > 2.741
B vs A1	A1	43	5	38	Reference				
	B	72	5	67	1.015	No effect	HR < 0.849; HR > 1.177	HR < 0.594; HR > 1.685	HR < 0.385; HR > 2.597
A2 vs B	A2	58	4	54	0.725	Small	HR < 0.801; HR > 1.249	HR < 0.51; HR > 1.961	HR < 0.309; HR > 3.241
	B	72	5	67	Reference				
**Disease-Free Interval**									
A1 vs A3	A1	38	4	34	3.681	Medium	HR < 0.745; HR > 1.342	HR < 0.431; HR > 2.32	HR < 0.245; HR > 4.079
	A3	79	2	77	Reference				
A2 vs A3	A2	46	6	40	6.407	Large	HR < 0.768; HR > 1.302	HR < 0.462; HR > 2.165	HR < 0.269; HR > 3.717

Comparison	Subpopulation	N	N_e	N_c	HR	HR effect	HR allocation adjusted critical value		
							$\alpha = 0.1$	$\alpha = 0.3$	$\alpha = 0.5$
							small effect	medium effect	large effect
	A3	79	2	77	Reference				
B vs A3	A3	79	2	77	Reference				
	B	64	5	59	4.291	Large	HR < 0.801; HR > 1.248	HR < 0.511; HR > 1.958	HR < 0.309; HR > 3.234
A2 vs A1	A1	38	4	34	Reference				
	A2	46	6	40	1.803	Medium	HR < 0.831; HR > 1.203	HR < 0.561; HR > 1.783	HR < 0.354; HR > 2.826
B vs A1	A1	38	4	34	Reference				
	B	64	5	59	1.094	No effect	HR < 0.85; HR > 1.177	HR < 0.594; HR > 1.683	HR < 0.386; HR > 2.594
A2 vs B	A2	46	6	40	1.751	Small	HR < 0.79; HR > 1.266	HR < 0.494; HR > 2.025	HR < 0.295; HR > 3.391
	B	64	5	59	Reference				
**Progression-Free Interval**									
A1 vs A3	A1	44	7	37	2.361	Medium	HR < 0.751; HR > 1.331	HR < 0.439; HR > 2.276	HR < 0.251; HR > 3.977
	A3	87	5	82	Reference				
A2 vs A3	A2	61	9	52	3.056	Medium	HR < 0.788; HR > 1.27	HR < 0.49; HR > 2.04	HR < 0.292; HR > 3.426
	A3	87	5	82	Reference				
B vs A3	A3	87	5	82	Reference				
	B	72	8	64	3.098	Medium	HR < 0.803; HR > 1.245	HR < 0.514; HR > 1.946	HR < 0.312; HR > 3.208
A2 vs A1	A1	44	7	37	Reference				
	A2	61	9	52	1.231	Small	HR < 0.839; HR > 1.191	HR < 0.575; HR > 1.738	HR < 0.367; HR > 2.721
B vs A1	A1	44	7	37	Reference				
	B	72	8	64	1.115	No effect	HR < 0.848; HR > 1.179	HR < 0.592; HR > 1.69	HR < 0.383; HR > 2.611
A2 vs B	A2	61	9	52	1.062	No effect	HR < 0.805; HR > 1.242	HR < 0.517; HR > 1.934	HR < 0.314; HR > 3.18
	B	72	8	64	Reference				

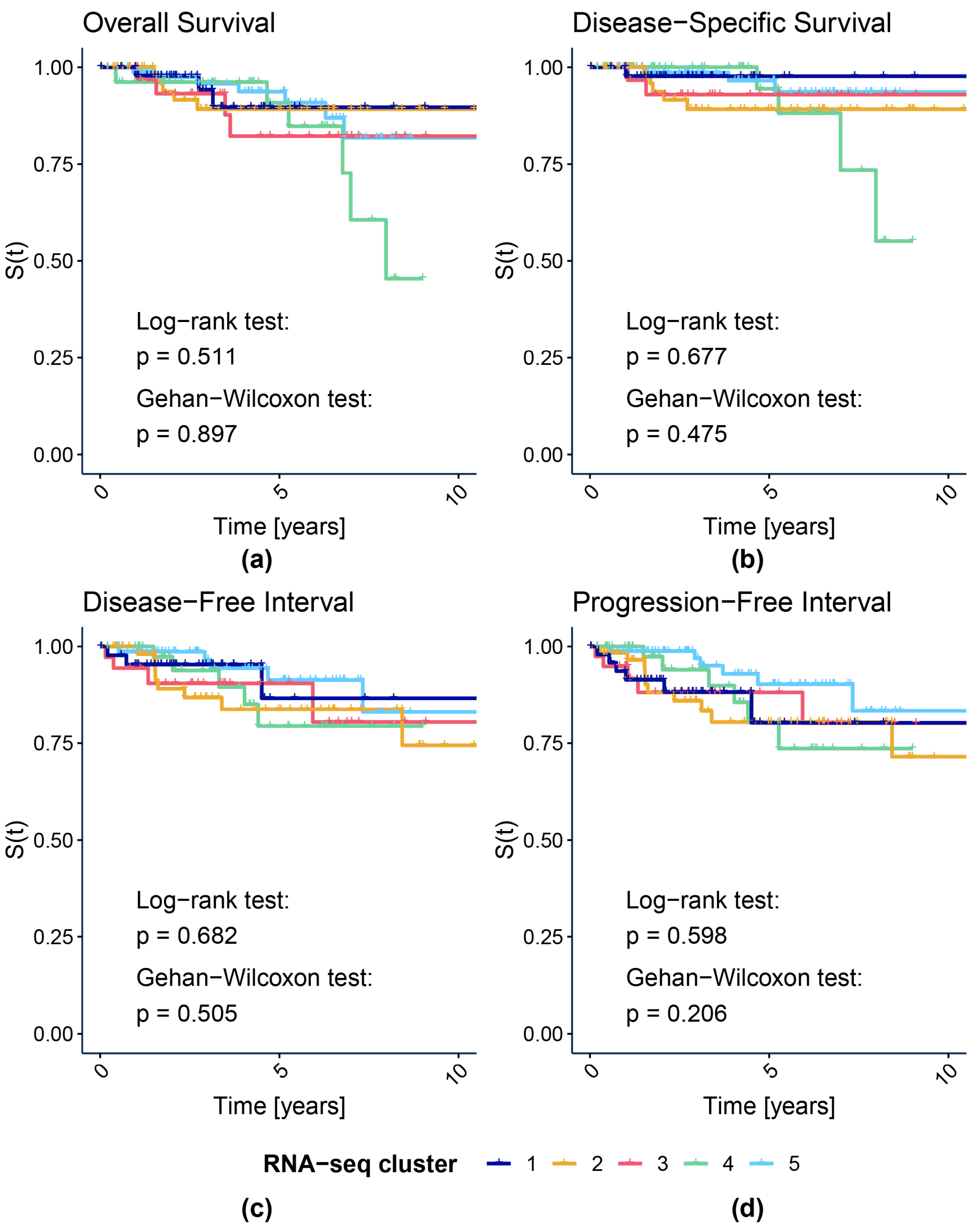

Figure S6. Kaplan-Meier survival curves of the clusters obtained based on the RNA sequencing results [30]. (**a**) Overall Survival; (**b**) Disease-Specific Survival; (**c**) Disease-Free Interval; (**d**) Progression-Free Interval.

Table S10. Association between categorical demographic and clinical factors and all subtypes identified with k-means clustering or based on PAM50 classifier.

Feature	χ^2	p-value	Cramér's V	Cramér's V effect threshold		
				Small	Medium	Large
**Proteomics-based subpopulations**						
**Race**	29.13	0.0155	0.1661	0.0577	0.1732	0.2887
**Ethnicity**	1.14	0.9508	0.0609	0.1	0.3	0.5
**AJCC Stage**	29.32	0.0146	0.1557	0.0577	0.1732	0.2887

<table>
<tr><th rowspan="2">Feature</th><th rowspan="2">χ^2</th><th rowspan="2">p-value</th><th rowspan="2">Cramér's V</th><th colspan="3">Cramér's V effect threshold</th></tr>
<tr><th>Small</th><th>Medium</th><th>Large</th></tr>
<tr><td>AJCC Tumor</td><td>29.33</td><td>0.0146</td><td>0.1554</td><td rowspan="2"></td><td rowspan="2"></td><td rowspan="2"></td></tr>
<tr><td>AJCC Node</td><td>28.79</td><td>0.0171</td><td>0.1538</td></tr>
<tr><td>AJCC Tumor Binarized</td><td>18.72</td><td>0.0022</td><td>0.2150</td><td rowspan="6">0.1</td><td rowspan="6">0.3</td><td rowspan="6">0.5</td></tr>
<tr><td>AJCC Node Binarized</td><td>13.09</td><td>0.0225</td><td>0.1796</td></tr>
<tr><td>AJCC Metastasis</td><td>2.58</td><td>0.7649</td><td>0.0798</td></tr>
<tr><td>Radiotherapy</td><td>5.45</td><td>0.3634</td><td>0.1157</td></tr>
<tr><td>Chemotherapy</td><td>19.39</td><td>0.0016</td><td>0.2183</td></tr>
<tr><td>Hormone Therapy</td><td>69.76</td><td><0.0001</td><td>0.4140</td></tr>
<tr><td colspan="7">PAM50-based subtypes</td></tr>
<tr><td>Race</td><td>34.68</td><td>0.0001</td><td>0.1812</td><td>0.0577</td><td>0.1732</td><td>0.2887</td></tr>
<tr><td>Ethnicity</td><td>4.09</td><td>0.2514</td><td>0.1155</td><td>0.1</td><td>0.3</td><td>0.5</td></tr>
<tr><td>AJCC Stage</td><td>20.13</td><td>0.0172</td><td>0.1290</td><td rowspan="3">0.0577</td><td rowspan="3">0.1732</td><td rowspan="3">0.2887</td></tr>
<tr><td>AJCC Tumor</td><td>24.28</td><td>0.0039</td><td>0.1414</td></tr>
<tr><td>AJCC Node</td><td>13.42</td><td>0.1447</td><td>0.1049</td></tr>
<tr><td>AJCC Tumor Binarized</td><td>19.58</td><td>0.0002</td><td>0.2199</td><td rowspan="6">0.1</td><td rowspan="6">0.3</td><td rowspan="6">0.5</td></tr>
<tr><td>AJCC Node Binarized</td><td>8.28</td><td>0.0406</td><td>0.1428</td></tr>
<tr><td>AJCC Metastasis</td><td>2.78</td><td>0.4263</td><td>0.0829</td></tr>
<tr><td>Radiotherapy</td><td>1.18</td><td>0.7572</td><td>0.0539</td></tr>
<tr><td>Chemotherapy</td><td>8.83</td><td>0.0316</td><td>0.1473</td></tr>
<tr><td>Hormone Therapy</td><td>62.85</td><td><0.0001</td><td>0.3930</td></tr>
</table>

Table cells with Cramér's V values are colored based on the effect size interpretation. Cramér's V effect interpretation thresholds were adjusted for the size of corresponding contingency tables.

Table S11. Summary of demographic and clinical categorical data.

Feature	Number of patients with records	Percentage of available records	Number of categories
**Race**	352	86.49%	4/3*
**Ethnicity**	307	75.43%	2
**AJCC Cancer Stage**	403	99.02%	4
**AJCC T**	405	99.51%	4
**AJCC T binarized**	405	99.51%	2
**AJCC N**	406	99.75%	4
**AJCC N binarized**	406	99.75%	2
**AJCC M**	405	99.51%	2

* The number of race categories was equal to 4 for all subtypes considered and 3 for luminal or luminal A subpopulations only.

Table S12. The list of proteins with at least a large η^2 effect between the identified four luminal subpopulations.

Selected proteins
14-3-3_beta-R-V
14-3-3_epsilon-M-C
ACVRL1-R-C
ADAR1-M-V
Akt_pS473-R-V
Akt_pT308-R-V
Annexin-1-M-E
Annexin_VII-M-V
ASNS-R-V
Bcl2A1-R-V
beta-Catenin-R-V
Bid-R-C
BRD4-R-V
c-Kit-R-V
c-Met-M-E
C-Raf-R-V
Caspase-8-M-E
Caveolin-1-R-V
CD20-R-C
cIAP-R-V
Claudin-7-R-V
COG3-R-V
Collagen_VI-R-V
Cyclin_B1-R-V
Cyclin_D1-R-V
DIRAS3-M-E
DUSP4-R-V
Dvl3-R-V
E-Cadherin-R-V
eEF2K-R-V
EGFR_pY1173-R-V
eIF4G-R-C
ER-alpha-R-V
ER-alpha_pS118-R-V
ETS-1-R-V
FoxM1-R-V
FOXO3a-R-C
GATA3-M-V
GSK3-alpha-beta-M-V
Heregulin-R-V
HSP70-R-C
Jak2-R-V
Ku80-R-C

Selected proteins
Lck-R-V
mTOR-R-V
MYH11-R-V
N-Cadherin-R-V
N-Ras-M-V
NDRG1_pT346-R-V
Notch1-R-V
p70S6K-R-V
PDCD4-R-C
PDK1_pS241-R-V
PKC-alpha-M-V
PR-R-V
PREX1-R-E
Raptor-R-V
Rb-M-E
Rb_pS807_S811-R-V
RBM15-R-V
S6-R-E
SCD-M-V
Src_pY527-R-V
STAT3_pY705-R-V
Stathmin-R-V

Table S13. The list of 40 proteins selected with the Gaussian-mixture-model-based feature filtration and with at least a large η^2 effect between the identified four luminal subpopulations.

Selected proteins
ADAR1-M-V
Akt_pS473-R-V
Akt_pT308-R-V
Annexin-1-M-E
ASNS-R-V
Bcl2A1-R-V
beta-Catenin-R-V
BRD4-R-V
c-Kit-R-V
Caveolin-1-R-V
Claudin-7-R-V
COG3-R-V
Collagen_VI-R-V
Cyclin_B1-R-V
Cyclin_D1-R-V
DUSP4-R-V
E-Cadherin-R-V
eEF2K-R-V

**Selected proteins**
eIF4G-R-C
ER-alpha-R-V
ER-alpha_pS118-R-V
FoxM1-R-V
GATA3-M-V
GSK3-alpha-beta-M-V
HSP70-R-C
Ku80-R-C
Lck-R-V
mTOR-R-V
MYH11-R-V
NDRG1_pT346-R-V
p70S6K-R-V
PDCD4-R-C
PR-R-V
PREX1-R-E
Rb_pS807_S811-R-V
RBM15-R-V
S6-R-E
SCD-M-V
Src_pY527-R-V
STAT3_pY705-R-V
